# Routine first‐trimester pre‐eclampsia screening and risk of preterm birth

**DOI:** 10.1002/uog.24915

**Published:** 2022-06-22

**Authors:** V. Giorgione, O. Quintero Mendez, A. Pinas, W. Ansley, B. Thilaganathan

**Affiliations:** ^1^ Fetal Medicine Unit St George's University Hospitals NHS Foundation Trust London UK; ^2^ Vascular Biology Research Centre Molecular and Clinical Sciences Research Institute, St George's University of London London UK

**Keywords:** first‐trimester pregnancy, iatrogenic preterm birth, pre‐eclampsia, preterm birth, spontaneous preterm birth, uteroplacental circulation

## Abstract

**Objectives:**

Preterm birth (PTB) is a major public health problem worldwide. It can occur spontaneously or be medically indicated for obstetric complications, such as pre‐eclampsia (PE) or fetal growth restriction. The main objective of this study was to investigate whether there is a shared uteroplacental etiology in the first trimester of pregnancy across PTB subtypes.

**Methods:**

This was a retrospective cohort study of singleton pregnancies that underwent screening for preterm PE as part of their routine first‐trimester ultrasound assessment at a tertiary center in London, UK, between March 2018 and December 2020. Screening for preterm PE was performed using the Fetal Medicine Foundation algorithm, which includes maternal factors, mean arterial pressure (MAP), uterine artery pulsatility index (UtA‐PI) and pregnancy‐associated plasma protein‐A (PAPP‐A). Women with a risk of ≥ 1 in 50 for preterm PE were classified as high risk and offered prophylactic aspirin (150 mg once a day) and serial ultrasound assessments. The following delivery outcomes were evaluated: PTB < 37 weeks, iatrogenic PTB (iPTB) and spontaneous PTB (sPTB). Logistic regression analyses were performed to assess the association of PTB, iPTB and sPTB with an increased risk of preterm PE. A model for prediction of PTB < 37 weeks and < 33 weeks was developed and its performance was compared with that of an existing model in the literature.

**Results:**

A total of 11 437 women were included in the study, of whom 475 (4.2%) had PTB. Of these, 308 (64.8%) were sPTB and 167 (35.2%) were iPTB. Patients with PTB had a higher body mass index, were more likely to be of black or Asian ethnicity, be smokers, have pregestational hypertension or diabetes, or have a history of previous PTB. They also had higher MAP (87.7 *vs* 86.0 mmHg, *P* < 0.0001), higher UtA‐PI multiples of the median (MoM) (0.99 *vs* 0.92, *P* < 0.0001) and lower PAPP‐A MoM (0.89 *vs* 1.08, *P* < 0.0001) compared to women with a term birth. In women at high risk of PE, the odds ratio for iPTB was 6.0 (95% CI, 4.29–8.43; *P* < 0.0001) and that for sPTB was 2.0 (95% CI, 1.46–2.86; *P* < 0.0001). A prediction model for PTB < 37 weeks and < 33 weeks, developed based on this cohort, included previous PTB, black ethnicity, chronic hypertension, diabetes mellitus, PAPP‐A MoM and UtA‐PI MoM. The performance of the model was similar to that of an existing first‐trimester prediction model for PTB < 33 weeks (area under the curve, 0.704 (95% CI, 0.653–0.754) *vs* 0.694 (95% CI, 0.643–0.746)).

**Conclusions:**

Increased first‐trimester risk for uteroplacental dysfunction was associated with both iPTB and sPTB, implying a shared etiological pathway. The same factors used to predict PE risk show acceptable discrimination to predict PTB at < 33 weeks. Women at high risk of uteroplacental dysfunction may warrant additional monitoring and management for an increased risk of sPTB. © 2022 The Authors. Ultrasound in Obstetrics & Gynecology published by John Wiley & Sons Ltd on behalf of International Society of Ultrasound in Obstetrics and Gynecology.

## Abstract 2

This article's abstract has been translated into Spanish and Chinese. Follow the links from the abstract to view the translations.

## RESUMEN

**Cribado habitual de la preeclampsia en el primer trimestre y riesgo de parto pretérmino**

**Objetivos:**

*El parto pretérmino (PPT) es un importante problema de salud pública en todo el mundo. Puede ocurrir de forma espontánea o estar indicado médicamente a causa de complicaciones obstétricas, como la preeclampsia (PE) o la restricción del crecimiento fetal. El objetivo principal de este estudio fue investigar si existe una etiología uteroplacentaria compartida en el primer trimestre del embarazo entre los subtipos de PPT*.

**Métodos:**

*Este fue un estudio de cohorte retrospectivo de embarazos con feto único que se sometieron a la detección de la PE pretérmino como parte de su evaluación rutinaria por ecografía en el primer trimestre en un centro terciario en Londres (Reino Unido) entre marzo de 2018 y diciembre de 2020. El cribado de la PE prematura se realizó mediante el algoritmo de la Fundación para la Medicina Fetal, que incluye factores maternos, presión arterial media (PAM), índice de pulsatilidad de la arteria uterina (UtA‐PI) y proteína plasmática A asociada al embarazo (PAPP‐A). Las mujeres con riesgo de ≥1 en 50 de sufrir una PE pretérmino fueron clasificadas como de alto riesgo y se les ofreció aspirina profiláctica (150 mg una vez al día) y evaluaciones ecográficas en serie. Se evaluaron los siguientes resultados del parto: PPT <37 semanas, PPT iatrogénico (iPPT) y PPT espontáneo (sPPT). Se realizaron análisis de regresión logística para evaluar la asociación de PPT, iPPT y sPPT con un mayor riesgo de PE prematura. Se comparó un modelo de predicción de la PPT <37 semanas y <33 semanas y su comportamiento con el de un modelo existente en la literatura*.

**Resultados:**

*Se incluyeron en el estudio 11.437 mujeres, de las cuales 475 (4,2%) tuvieron un PPT. De ellos, 308 (64,8%) fueron sPPT y 167 (35,2%) iPPT. Las pacientes con PPT tenían un mayor índice de masa corporal, eran más propensas a ser de etnia negra o asiática, ser fumadoras, tener hipertensión pregestacional o diabetes, o tener un historial previo de PPT. También tenían una PAM más alta (87,7 frente a 86,0 mmHg, P<0.0001), mayores múltiplos de la mediana (MdM) de la UtA‐PI (0,99 frente a 0,92, P<0.0001) y menores MdM de la PAPP‐A (0,89 frente a 1,08, P<0.0001) en comparación con las mujeres con un parto a término. En las mujeres con alto riesgo de PE, la razón de momios para la iPPT fue de 6,0 (IC 95%, 4,29–8,43) P<0.0001) y para la sPPT fue de 2,0 (IC 95%, 1,46–2,86 P<0.0001). Un modelo de predicción para el PPT<37 semanas y <33 semanas, desarrollado sobre la base de esta cohorte, incluyó el PPT previo, la etnia negra, la hipertensión crónica, la diabetes mellitus, la MdM de la PAPP‐A y la MdM de la UtA‐PI. El rendimiento del modelo fue similar al de un modelo de predicción del primer trimestre existente para el PPT <33 semanas (área bajo la curva, 0,704 (IC 95%, 0,653–0,754) frente a 0,694 (IC 95%, 0,643–0,746))*.

**Conclusiones:**

*El aumento del riesgo de disfunción uteroplacentaria en el primer trimestre se asoció tanto a la iPPT como a la sPPT, lo que implica una vía etiológica compartida. Los mismos factores utilizados para predecir el riesgo de PE muestran una discriminación aceptable para predecir el PPT <33 semanas. Las mujeres con alto riesgo de disfunción uteroplacentaria pueden justificar una vigilancia y tratamiento adicionales para un mayor riesgo de sPPT*.

## 摘要

**怀孕早期先兆子痫常规筛查和早产风险**

**目的:**

早产(PTB)是世界范围内的重大公共卫生问题。 它可以自发发生，也可以在医学上用于产科并发症，例如先兆子痫 (PE) 或胎儿生长受限。 本研究的主要目的是调查 PTB 亚型在妊娠前三个月是否存在共同的子宫胎盘病因。

**方法:**

这是一项对单胎妊娠的回顾性队列研究，这些孕妇于 2018 年 3 月至 2020 年 12 月在英国伦敦的一家三级孕检中心接受早产先兆子痫( PE )筛查作为其孕早期常规超声评估的一部分。使用胎儿医学基金会算法进行早产先兆子痫( PE) 筛查，该算法包括母体因素、平均动脉压 (MAP)、子宫动脉搏动指数 (UtA‐PI) 和妊娠相关血浆蛋白 A (PAPP‐A)。早产 先兆子痫(PE) 风险≥1/50 的女性被归类为高风险，并提供预防性阿司匹林(150 mg，每天一次)和系列超声评估。并对以下分娩结果进行了评估:早产(PTB)<37 周、医源性早产 (iPTB) 和自发性早产 (sPTB)。进行逻辑回归分析以评估 PTB、iPTB 和 sPTB 与早产 先兆子痫(PE) 风险增加的关联。一个用于预测 PTB <37 周和 <33 周的模型得到了开发，并将其性能与文献中现有模型的性能进行了比较。

**结果:**

共计有11 437名女性纳入研究，其中475人(4.2%)是早产，而这些早产孕妇中，308 例(64.8%)为 自发性早产(sPTB)，167 例(35.2%)为医源性早产( iPTB)。 早产(PTB) 患者的体重指数较高， 黑人或亚裔，吸烟者，有妊娠前高血压或糖尿病，或既往 PTB 病史的患者几率更高。他们的平均动脉压( MAP)更高(87.7 对比86.0 mmHg，P<0.0001)，更高的子宫动脉搏动指数 ( UtA‐PI) 中位数倍数(MoM)(0.99 对 0.92，P<0.0001)和更低的和妊娠相关血浆蛋白 A (PAPP‐A )MoM(0.89 对 1.08，P<0.0001 ) 与足月分娩的女性相比。在 先兆子痫(PE) 高风险女性中，医源性早产(iPTB) 的优势比为 6.0(95% CI，4.29‐8.43；P<0.0001)，自发性早产(sPTB) 的优势比为 2.0(95% CI，1.46‐2.86；P<0.0001)。基于该队列开发的早产(PTB)<37 周和 <33 周的预测模型包括既往早产史( PTB)、黑人种族、慢性高血压、糖尿病、和妊娠相关血浆蛋白 A (PAPP‐A )中位数倍数(MoM) 和子宫动脉搏动指数(UtA‐PI )中位数倍数(MoM)。该模型的性能类似于现有的孕早期预测模型，用于 PTB<33 周(曲线下面积，0.704(95% CI，0.653–0.754)对比0.694(95% CI，0.643–0.746)) .

**结论:**

妊娠早期子宫胎盘功能障碍风险增加与医源性早产( iPTB )和 自发性早产(sPTB) 相关，这意味着共同的病因途径。用于预测先兆子痫( PE )风险的相同因素显示了在 <33 周时预测 PTB 的可接受的区别。子宫胎盘功能障碍高风险的女性可能需要对增加的自发性早产(sPTB)风险进行观察和管理。

**CONTRIBUTION**

**What are the novel findings of this work?**

Women who are classified in the first trimester as being at high risk of developing preterm pre‐eclampsia are also at increased risk of both iatrogenic and spontaneous preterm birth (PTB). Our model for prediction of PTB < 33 weeks' gestation showed acceptable discrimination, similar to that obtained by an existing PTB prediction model.

**What are the clinical implications of this work?**

Women with iatrogenic and spontaneous PTB share signs of uteroplacental dysfunction in the first trimester that may be evaluated by screening for preterm pre‐eclampsia using the Fetal Medicine Foundation algorithm. Women identified in the first trimester as being at high risk of preterm pre‐eclampsia may benefit from measures to assess and reduce the risk of spontaneous PTB.

## Introduction

Preterm birth (PTB) affects between 5% and 18% of pregnancies. The resulting complications are the leading cause of death in children under 5 years old, and are a major public health problem worldwide^1^, ^2^. Maternal or fetal complications such as pre‐eclampsia (PE), fetal growth restriction (FGR), placenta previa or placental abruption are significant reasons for iatrogenic PTB (iPTB), which accounts for 20–30% of all instances of PTB^3^, ^4^. However, most PTBs occur spontaneously (sPTB), with or without preterm prelabor rupture of membranes (PPROM).

Multiple putative mechanisms have been proposed for sPTB^2^, one of which is uteroplacental malperfusion associated with the development of PE and FGR^5^, ^6^, ^7^. Placental lesions consistent with maternal vascular hypoperfusion have been found in pregnancies complicated by preterm labor^8^, ^9^. An imbalance of angiogenic factors, with increased levels of antiangiogenic elements such as soluble endoglin and soluble fms‐like tyrosine kinase‐1 (sFlt‐1) has also been associated with sPTB and early‐term birth^10^, ^11^.

The Aspirin for Evidence‐Based Pre‐eclampsia Prevention (ASPRE) study introduced a first‐trimester screening algorithm for preterm PE that combines maternal cardiovascular and placental factors to recognize women at higher risk of early uteroplacental dysfunction^12^. A subsequent clinical‐effectiveness study demonstrated that prophylactic treatment with low‐dose aspirin in women at high risk of preterm PE reduced the incidence of preterm PE by 80%^12^, ^13^. In addition to a reduction in iPTB, it was found that low‐dose aspirin prophylaxis also reduced sPTB in women at risk of PE by 7% at < 37 weeks and 14% at < 34 weeks^14^. These findings are consistent with previous studies in which both types of PTB were associated with low pregnancy‐associated plasma protein‐A (PAPP‐A) and increased uterine artery Doppler pulsatility index (UtA‐PI) in the first trimester^15^, ^16^.

The main objective of this study was to determine whether there is a shared uteroplacental etiology in the first trimester of pregnancy across PTB subtypes. Therefore, we investigated the risk factors for PTB in a large cohort of patients who underwent first‐trimester screening for preterm PE, and the risk of sPTB in the group at high risk for preterm PE. Moreover, we validated an existing prediction model for PTB that is based on first‐trimester biomarkers^16^.

## Methods

### Population

This was a retrospective cohort study of all singleton pregnancies booked for routine first‐trimester ultrasound assessment at < 14 weeks' gestation at St George's Hospitals NHS Foundation Trust, London, UK, between March 2018 and December 2020. All pregnancies underwent first‐trimester screening for preterm PE according to the Fetal Medicine Foundation algorithm which includes maternal factors, mean arterial pressure (MAP), UtA‐PI and PAPP‐A. It has been demonstrated previously that PAPP‐A performs similarly to placental growth factor (PlGF) in detecting preterm PE when applied in the same clinical setting as that of the current study^17^. At the routine 11–13‐week ultrasound scan, crown–rump length was used to establish gestational age, and the UtA‐PI and MAP were measured using established protocols^18^, ^19^, ^20^. Those with a risk of ≥ 1 in 50 for preterm PE were classified as being at high risk and offered prophylactic aspirin 150 mg once a day, as described in previous studies^13^, ^21^. In addition, they were scheduled for growth scans at 28 and 36 weeks' gestation and offered labor induction from 40 weeks. All women who delivered at St George's University Hospitals NHS Foundation Trust and who were managed using this protocol were included in the analysis. Exclusion criteria included pregnancies resulting in miscarriage or termination and those lost to follow‐up.

### Study variables and outcomes

Maternal demographic characteristics, past medical history and prenatal data were obtained from the hospital's ultrasound database (ViewPoint version 5.6.26.148, ViewPoint Bildverarbeitung GmbH, Wessling, Germany) and delivery outcomes (gestational age, birth weight and type of labor and delivery) were obtained from the maternity birth registry (EuroKing, Wellbeing Software, Mansfield, UK). Both databases are routinely used and checked for the provision of healthcare. The primary outcome was PTB, defined as delivery at < 37 weeks' gestation. The secondary outcomes were iPTB, defined as PTB resulting from indicated induction of labor or Cesarean section because of fetal and/or maternal complications, and sPTB due to spontaneous preterm labor or PPROM.

This study was conducted as part of a local clinical audit, and patients' identifiable information was removed before merging the datasets. The local ethics committee advised that formal ethical approval was not required for this retrospective study.

### Statistical analysis

Categorical data are presented as *n* (%), and compared using Fisher's exact test or chi‐square test. Variables were assessed for normality by the Shapiro–Wilk test and by visualizing the histograms. Continuous data were not normally distributed and were therefore presented as median with interquartile range (IQR). Non‐parametric analysis using the Mann–Whitney *U*‐test was performed to compare continuous data between the study groups. Logistic regression analyses were performed to assess the association of PTB, iPTB and sPTB with increased risk of preterm PE. Univariate and multivariate logistic regression analyses were performed, adjusting for risk factors for PTB.

Equations reported by Stout *et al*. for the full logistic regression model (comprising placental protein‐13 (PP13), PAPP‐A, mean UtA‐PI, black ethnicity, chronic hypertension, diabetes and prior PTB) for PTB at < 37 weeks and < 33 weeks were applied to our dataset to obtain predicted risk for each individual^16^. As PP13 was not available in our cohort, multiples of the median (MoM) equal to 1 was used for PP13 in the equation. Moreover, a binomial logistic regression was performed to ascertain the effect of predictors from the pre‐existing model (previous PTB, black ethnicity, chronic hypertension, diabetes, PAPP‐A multiples of the median (MoM) and UtA‐PI MoM) on the likelihood that participants have PTB in our cohort^16^. Discrimination of the model (i.e. its ability to distinguish between delivering preterm and delivering at term) was assessed using the area under the curve (AUC) with 95% CI. An AUC of 0.50 indicates no discriminative ability, and the closer the AUC is to 1.0, the better the discriminative performance. Calibration was assessed graphically using a calibration plot, with a diagonal line implying perfect calibration^22^. *P*‐values < 0.05 were considered statistically significant. The statistical analysis was performed using SPSS version 27.0 (SPSS Inc., Chicago, IL, USA).

## Results

**Figure 1:**
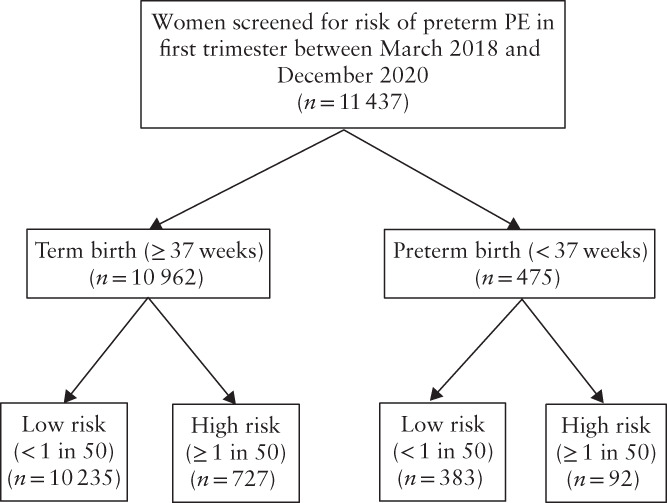
Risk of preterm pre‐eclampsia (PE) in women included in study cohort, according to whether they delivered at term or preterm.

During the study period, 11 437 women were screened for preterm PE in the first trimester using the FMF algorithm. Of these women, 475 (4.2%) had a preterm birth, of which 308 (64.8%) were sPTB and 167 (35.2%) were iPTB (Figure 1). Maternal demographic characteristics, history of PTB and data from the first‐trimester screening assessment are shown in Table 1. Women who delivered preterm, compared with those delivering at term, had a higher BMI at booking, were more likely to be of black or Asian ethnicity, be smokers, have pregestational hypertension or diabetes, or have a history of PTB. Women who delivered preterm, compared with those delivering at term, were also more likely to be assessed as being at high risk for preterm PE (19.4% *vs* 6.6%, *P* < 0.0001), with significantly higher MAP (87.7 *vs* 86.0, *P* < 0.0001), higher UtA‐PI MoM (0.99 *vs* 0.92, *P* < 0.0001) and lower PAPP‐A MoM (0.89 *vs* 1.08, *P* < 0.0001). The results of univariate and multivariate binary logistic regression analyses with PTB at < 37 weeks as the main outcome are shown in Table S1.

**Table 1:** Maternal and pregnancy‐related characteristics of women with a singleton pregnancy, according to whether they delivered at term or had preterm birth (PTB)

Characteristic	Term birth (*n* = 10 962)	PTB (*n* = 475)	*P*
Maternal age (years)	32 (29–35)	32 (29–36)	0.714
Weight (kg)	65.5 (58.6–75.0)	67.9 (59.1–78.7)	0.015
BMI (kg/m^2^)	24.2 (21.8–27.6)	25.6 (22.1–29.3)	< 0.0001
Ethnicity			
White	7313 (66.7)	249 (52.4)	< 0.0001
Black	1167 (10.6)	91 (19.2)	< 0.0001
Asian	2014 (18.4)	112 (23.6)	0.002
Mixed/other	468 (4.3)	23 (4.8)	0.787
History of PTB	614 (5.6)	81 (17.1)	< 0.0001
Current smoker	439 (4.0)	32 (6.7)	0.003
Conception by ART	395 (3.6)	24 (5.1)	0.100
Diabetes mellitus	93 (0.8)	16 (3.4)	< 0.0001
Chronic hypertension	78 (0.7)	12 (2.5)	< 0.0001
MAP (mmHg)	86.0 (81.2–91.2)	87.7 (82.3–92.5)	< 0.0001
UtA‐PI MoM	0.92 (0.74–1.12)	0.99 (0.77–1.24)	< 0.0001
PAPP‐A MoM	1.08 (0.75–1.52)	0.89 (0.61–1.32)	< 0.0001

Data are shown as median (interquartile range) or *n* (%).

ART, assisted reproductive technology; BMI, body mass index; MAP, mean arterial pressure; MoM, multiples of the median; PAPP‐A, pregnancy‐associated plasma protein‐A; UtA‐PI, uterine artery pulsatility index.

Maternal and pregnancy‐related characteristics in women with sPTB compared to those without are presented in Table 2. Asian ethnicity, history of PTB, higher UtA‐PI MoM and lower PAPP‐A MoM were significantly associated with sPTB (Table S2). These associations remained statistically significant in the multivariate logistic regression analysis, with odds ratios (OR) of 2.92 (95% CI, 2.12–4.02) for a history of PTB, 0.71 (95% CI, 0.57–0.87) for PAPP‐A MoM and 1.60 (95% CI, 1.08–2.37) for UtA‐PI MoM (Table S2). Women who had iPTB were older, had a higher BMI, were more likely to be of black ethnicity and had more comorbidities, including higher MAP at the first‐trimester visit, compared with pregnancies complicated by sPTB (Table S3). The indications for elective birth in the iPTB group are shown in Table S4.

**Table 2:** Maternal and pregnancy‐related characteristics of study population, according to whether they experienced spontaneous preterm birth (PTB)

Characteristic	Term birth or iatrogenic PTB (*n* = 11 129)	Spontaneous PTB (*n* = 308)	*P*
Maternal age (years)	32 (29–35)	32 (28–35)	0.122
BMI at screening (kg/m^2^)	24.2 (21.8–27.7)	25.0 (21.4–28.8)	0.157
Ethnicity			
White	7387 (66.4)	175 (56.8)	0.001
Black	1214 (10.9)	44 (14.3)	0.062
Asian	2050 (18.4)	76 (24.7)	0.005
Mixed/other	478 (4.3)	13 (4.2)	0.950
History of PTB	644 (5.8)	51 (16.6)	< 0.0001
Current smoker	455 (4.1)	16 (5.2)	0.335
Conception by ART	403 (3.6)	16 (5.2)	0.195
Diabetes mellitus	103 (0.9)	6 (1.9)	0.068
Chronic hypertension	86 (0.8)	4 (1.3)	0.309
UtA‐PI MoM	0.92 (0.74–1.13)	0.97 (0.76–1.23)	0.002
PAPP‐A MoM	1.08 (0.75–1.53)	0.90 (0.64–1.34)	< 0.0001

Data are shown as median (interquartile range) or *n* (%).

ART, assisted reproductive technology; BMI, body mass index; MoM, multiples of the median; PAPP‐A, pregnancy‐associated plasma protein‐A; UtA‐PI, uterine artery pulsatility index.

In women at high risk of preterm PE, there was a significant increase in the risk for both sPTB and iPTB (Table 3). The ORs in the high‐risk group for PTB < 37 weeks and < 33 weeks were 3.4 (95% CI, 2.66–4.30) and 2.3 (95% CI, 1.47–3.67), respectively.

Similarly, the ORs for iPTB and sPTB in the high‐risk group were 6.0 (95% CI, 4.29–8.43) and 2.0 (95% CI, 1.46–2.86), respectively.

**Table 3:** Risk of preterm birth (PTB) in women with high risk *vs* those with a low risk for preterm pre‐eclampsia (PE)

Parameter	Low risk for PE (< 1 in 50) (*n* = 10 618)	High risk for PE (≥ 1 in 50) (*n* = 819)	Odds ratio (95% CI)	*P*
All PTB < 37 weeks	383 (3.61)	92 (11.23)	3.4 (2.66–4.30)	< 0.0001
All PTB < 33 weeks	125 (1.18)	22 (2.69)	2.3 (1.47–3.67)	< 0.0001
Spontaneous PTB	267 (2.51)	41 (5.01)	2.0 (1.46–2.86)	< 0.0001
Iatrogenic PTB	116 (1.09)	51 (6.23)	6.0 (4.29–8.43)	< 0.0001

Data for the two risk groups are given as *n* (%).

**Figure 2:**
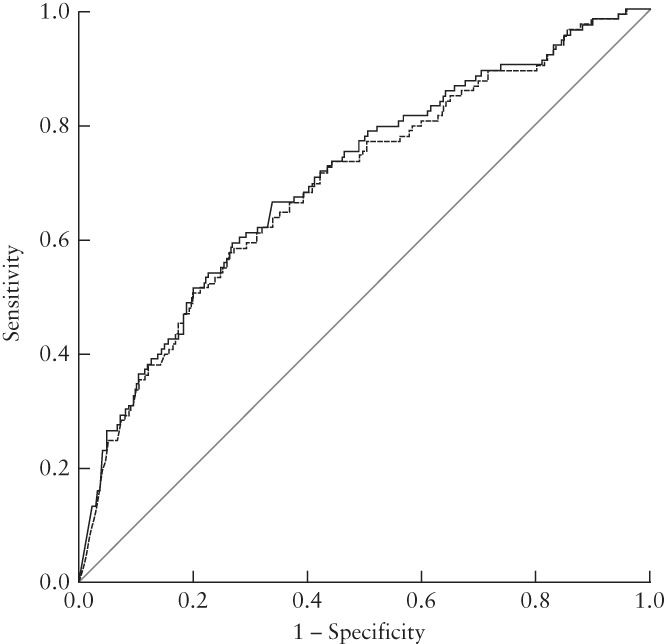
Receiver‐operating‐characteristics curves for prediction of preterm birth at < 33 weeks using model from current study (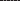) (previous preterm birth, black ethnicity, chronic hypertension, diabetes mellitus, pregnancy‐associated plasma protein‐A multiples of the median (MoM) and uterine artery pulsatility index MoM) and the prediction model of Stout *et al*.^16^ (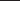).

The logistic regression model by Stout *et al*.^16^ for PTB at < 37 weeks (χ^2^(6) = 155.731, *P* < 0.0001) explained 5.0% of the variance in PTB (Nagelkerke's *R* ^2^), and correctly classified 95.7% of cases (sensitivity 3.2%; specificity 99.6%; positive predictive value 26.3%; negative predictive value 96.0%). All six predictors (previous PTB, black ethnicity, chronic hypertension, diabetes, PAPP‐A MoM and UtA‐PI MoM) were statistically significant in our cohort (Table S1) and were included in the model. The AUC values of our PTB prediction model were 0.654 (95% CI, 0.627–0.681) for delivery at < 37 weeks and 0.704 (95% CI, 0.653–0.754) for delivery at < 33 weeks, which were similar to those obtained using the model described by Stout *et al*.^16^ (AUC, 0.646 (95% CI, 0.619–0.673) for delivery < 37 weeks; AUC, 0.694 (95% CI, 0.643–0.746) for delivery < 33 weeks) (Figure 2). Figure S1 illustrates the calibration plot of the pre‐existing model described by Stout *et al*.^16^ for PTB at < 37 weeks, demonstrating overprediction of the risk. In a subanalysis of 6274 nulliparous women (i.e. excluding previous PTB as a predictor), our model and that of Stout *et al*. gave similar results, with AUC values of 0.638 (95% CI, 0.602–0.674) and 0.625 (95% CI, 0.588–0.662) for PTB at < 37 weeks, respectively. The corresponding AUC values for PTB at < 33 weeks were 0.712 (95% CI, 0.644–0.780) for our model and 0.684 (95% CI, 0.613–0.756) for the model developed by Stout *et al*.^16^.

## Discussion

**Figure 3:**
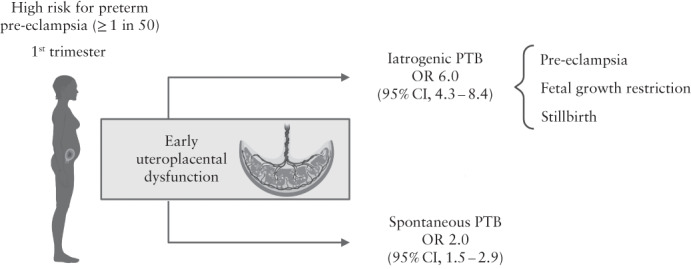
Early uteroplacental dysfunction in pathogenesis of iatrogenic and spontaneous preterm birth (PTB). OR, odds ratio.

The findings of the study confirm that women assessed as being at high risk for placental dysfunction in the first trimester were at increased risk of sPTB in addition to the expected increase in iPTB for pregnancy complications (Figure 3). The placental function markers used in our study were also able to replicate the performance of and externally validate an existing first‐trimester prediction model for PTB.

### Interpretation of study findings and comparison with published literature

PTB is a significant cause of neonatal mortality and morbidity, and is thus a substantial public health problem that the World Health Organization and the UK Department of Health aim to reduce substantially in the near future^23^, ^24^. The manifestations of early uteroplacental dysfunction (which include PE and FGR) result in more than half of all instances of iPTB^25^. Therefore, an effective screening program able to identify women at risk of placental dysfunction and reduce preterm PE by using low‐dose aspirin will also be beneficial in reducing the rate of iPTB^13^. Consistent with this hypothesis, our data showed that women categorized as having a high risk of preterm PE have a 6‐fold higher risk of iPTB and a 2‐fold higher risk of sPTB.

Women who delivered spontaneously preterm had lower PAPP‐A MoM and higher UtA‐PI MoM (recognized markers of placental insufficiency) in the first trimester, compared with those who did not. It is accepted that, while the final common pathway of labor onset may be the same, the etiology of sPTB is multifactorial, including factors such as infection/inflammation, placental ischemia, cervical weakness and uterine overdistension^2^. The role of uteroplacental ischemia in sPTB is supported by increasing evidence from placental histology and serum biomarkers^5^, ^6^, ^7^, ^8^, ^9^, ^10^. In sPTB, evidence of maternal vascular malperfusion of the placental bed was identified more often than inflammation/infection, and was further associated with severe adverse neonatal outcomes such as intraventricular hemorrhage^3^. Furthermore, approximately one‐third of patients with sPTB have a failure of physiological transformation of the myometrial segments of the spiral arteries, which is typically associated with PE and FGR^8^, ^9^, ^26^. Finally, preterm labor is associated with an antiangiogenic profile of an elevated ratio of sFlt‐1 to PlGF, in common with PE and FGR^10^, ^11^.

The ASPRE trial did not find a significant difference in sPTB in the group who received aspirin compared with the placebo group^12^. Nevertheless, the results of an individual participant data meta‐analysis comprising 17 randomized controlled trials with a total of 28 797 women showed a lower risk of sPTB at < 37 weeks (RR, 0.93; 95% CI, 0.86–0.996) and < 34 weeks (RR, 0.86; 95% CI, 0.76–0.99) in women assigned to antiplatelet treatment compared with placebo or no treatment^14^. On the basis of this finding, the authors suggested that prophylactic treatment with low‐dose aspirin could be used in a broader population of pregnant women than those with a high risk of placental dysfunction^14^.

Five prediction models for sPTB, including only maternal factors at < 16 weeks' gestation, were externally validated in a Dutch unselected cohort of 2614 women^27^. The AUC values for these models ranged from 0.54 to 0.67 for sPTB < 37 weeks and from 0.56 to 0.70 for sPTB at < 34 weeks. The models performed better in parous than in nulliparous women^27^. Signs of uteroplacental dysfunction, such as low PAPP‐A and increased UtA‐PI, in women who delivered preterm for iatrogenic or spontaneous reasons, have been reported in previous studies^15^, ^16^, ^28^. These factors may help improve prediction for women at risk of iPTB and sPTB, regardless of parity. Discrimination in our model for the prediction of PTB was similar, even in nulliparous women, to that obtained when using the existing model described by Stout *et al*.^16^, but the performance was much lower than the AUC values of 0.90–0.91 reported by the authors during model development and internal validation^16^. The underperformance may have resulted from model overfitting or the systematic treatment with 150 mg aspirin per day in our high‐risk group, which may have been protective against some iPTB and sPTB.

### Clinical and research implications

Our findings support the hypothesis that preterm labor may be initiated by early uteroplacental malperfusion in a proportion of women. This finding has raised the possibility that high‐risk women identified in early pregnancy could be offered cervical length/fetal fibronectin monitoring and risk‐reducing measures for sPTB^29^, ^30^. However, further studies are necessary to determine the mechanisms of the uteroplacental dysfunction observed in cases of both iPTB and sPTB, and to evaluate the best means to monitor and prevent sPTB in women at high risk of early uteroplacental insufficiency as determined using the FMF algorithm.

### Strengths and limitations

This study evaluates whether women screened and identified as being at high risk for preterm PE are also at increased risk of sPTB. Comparing maternal demographic and medical characteristics in a large cohort of patients is a strength of this study. Our model had moderate screening performance for PTB at < 33 weeks, achieving similar results to the existing model by Stout *et al*.^16^; however, their screening performance should be ameliorated before clinical implementation. Another significant limitation is the retrospective design of the study and the potential presence of confounding factors, as patients at high risk of preterm PE are likely to change their behavior to reduce this risk. These women also received prophylactic low‐dose aspirin up to 36 weeks' gestation, which is known to minimize preterm PE. Moreover, the study did not include the most severe preterm births with PPROM at < 24 weeks, resulting in second‐trimester miscarriage.

### Conclusions

Women identified in the first trimester as being at high risk for preterm PE should be considered at increased risk of sPTB as well as iPTB. Demographic, biochemical and biophysical data obtained in the first trimester to assess preterm PE risk may also be useful in assessing the risk of PTB. Further studies are necessary to improve PTB prediction in the first trimester, and to assess the best preventive strategy to reduce the risk of sPTB, such as assessment of cervical length at midgestation or other risk‐reducing measures.

## Supporting information


**Figure S1** Calibration plot of existing model for prediction of preterm birth (< 37 weeks) by Stout *et al*.^16^.

**Table S1** Univariate and multivariate analysis for risk of preterm birth (PTB)
**Table S2** Univariate and multivariate analysis for risk of spontaneous preterm birth (sPTB)
**Table S3** Comparison of maternal characteristics between women with spontaneous preterm birth (sPTB) and those with iatrogenic preterm birth (iPTB)
**Table S4** Indications for iatrogenic preterm birth (iPTB)

## Data Availability

The data that support the findings of this study are available from the corresponding author upon reasonable request.
