# Supplementary material for: Routine first‐trimester pre‐eclampsia screening and risk of preterm birth
Source: Ultrasound Obstet Gynecol. 2022 Jun 22;60(2):185–91. doi: 10.1002/uog.24915 (PMC9545360; doi:10.1002/uog.24915)
Supplement: Supplementary file 1 — Figure S1 Calibration plot of existing model for prediction of preterm birth (< 37 weeks) by Stout et al. 16 . [file UOG-60-185-s002.docx]

**Figure S1.** Calibration plot of an existing prediction model for the prediction of preterm birth (37 weeks).

**
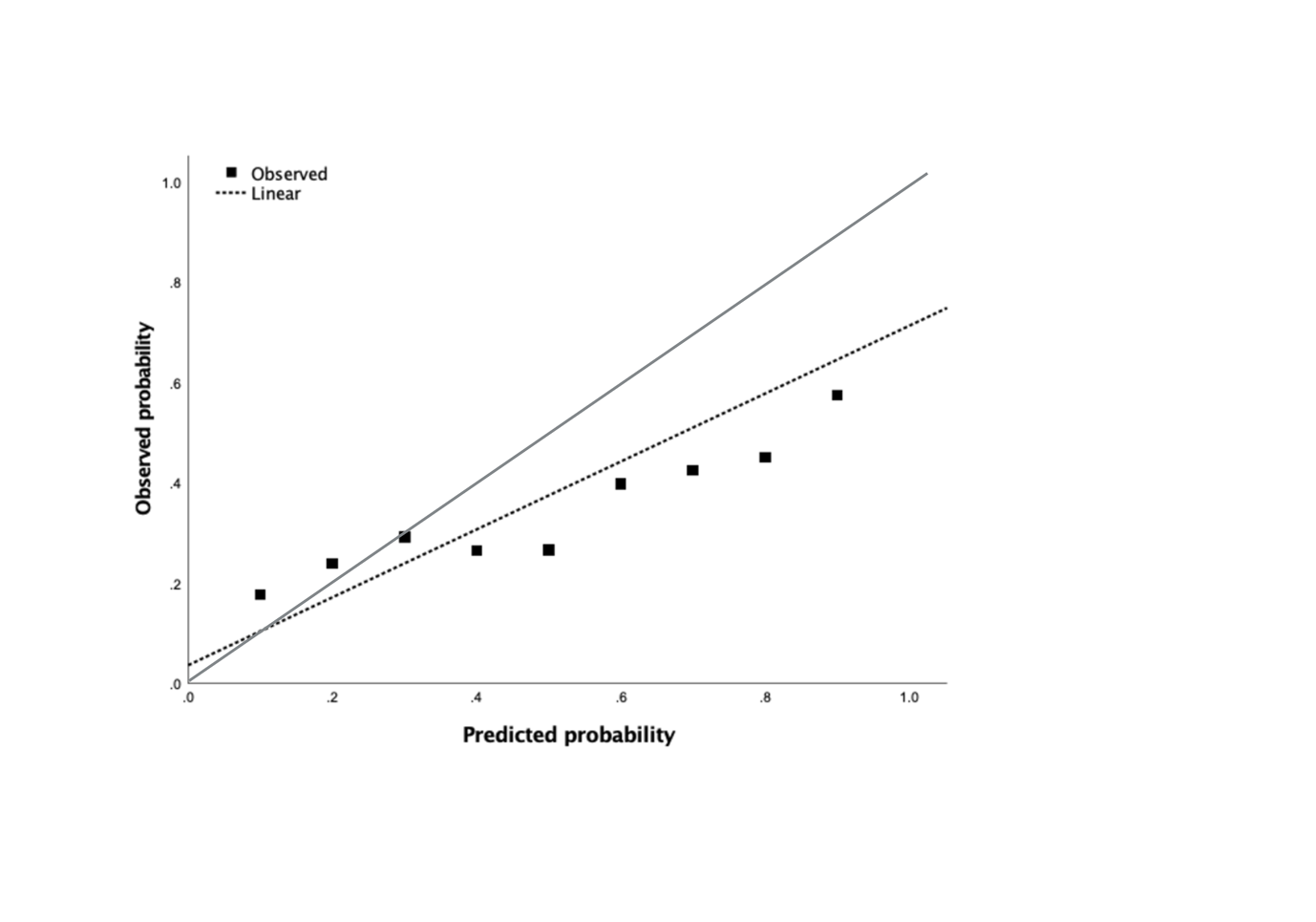
**
