# Supplementary material for: Routine first‐trimester pre‐eclampsia screening and risk of preterm birth
Source: Ultrasound Obstet Gynecol. 2022 Jun 22;60(2):185–91. doi: 10.1002/uog.24915 (PMC9545360; doi:10.1002/uog.24915)
Supplement: Supplementary file 2 — Table S1 Univariate and multivariate analysis for risk of preterm birth (PTB) Table S2 Univariate and multivariate analysis for risk of spontaneous preterm birth (sPTB) Table S3 Comparison of maternal characteristics between women with spontaneous preterm birth (sPTB) and those with iatrogenic preterm birth (iPTB) Table S4 Indications for iatrogenic preterm birth (iPTB) [file UOG-60-185-s001.docx]

**Table S1** Univariate and multivariate analysis for risk of preterm birth (PTB)

|  | **Univariate analysis** | | **Multivariate analysis** | |
| --- | --- | --- | --- | --- |
|  | **OR (95% CI)** | **P value** | **OR (95% CI)** | **P-value** |
| **BMI** | 1.00 (0.99-1.00) | 0.446 | - | - |
| **Ethnicity (Black)** | 1.99 (1.57-2.52) | **<0.0001** | 1.80 (1.41-2.31) | **<0.0001** |
| **Ethnicity (Asian)** | 1.37 (1.10-1.70) | **0.004** | - | **-** |
| **Previous PTB** | 3.47 (2.69-4.46) | **<0.0001** | 2.84 (1.18-3.71) | **<0.0001** |
| **Smoking** | 1.73 (1.20-2.51) | **0.004** | - | **-** |
| **Diabetes mellitus** | 4.08 (2.38-6.70) | **<0.0001** | 2.96 (1.66-5.30) | **<0.0001** |
| **Chronic hypertension** | 3.62 (1.96-6.69) | **<0.0001** | 2.25 (1.17-4.33) | **0.015** |
| **UtA-PI MoM** | 2.26 (1.66-3.09) | **<0.0001** | 1.95 (1.42-2.69) | **<0.0001** |
| **PAPP-A MoM** | 0.60 (0.50-0.71) | **<0.0001** | 0.66 (0.55-0.78) | **<0.0001** |

BMI body mass index, UtA-PI uterine artery pulsatility index, PAPP-A pregnancy-associated plasma protein A, MoM multiple of median

**Table S2** Univariate and multivariate analysis for risk of spontaneous preterm birth (PTB)

|  | **Univariate analysis** | | **Multivariate analysis** | |
| --- | --- | --- | --- | --- |
|  | **OR (95% CI)** | **P-value** | **OR (95% CI)** | **P-value** |
| **Ethnicity (Asian)** | 1.45 (1.12-1.89) | **0.006** | 1.37 (1.05-1.79) | **0.021** |
| **Previous PTB** | 3.23 (2.34-4.41) | **<0.0001** | 2.92 (2.12-4.02) | **<0.0001** |
| **UtA-PI MoM** | 1.94 (1.32-2.85) | **0.001** | **1.60 (1.08-2.37)** | **0.020** |
| **PAPP-A MoM** | 0.65 (0.53-0.80) | **<0.0001** | **0.71 (0.57-0.87)** | **0.001** |

UtA-PI uterine artery pulsatility index, PAPP-A pregnancy-associated plasma protein A, MoM multiple of median

**Table S3.** Comparison of maternal characteristics between women with spontaneous preterm birth (sPTB) and those with iatrogenic preterm birth (iPTB)

| **Variables** | | **sPTB n=308** | | **iPTB n=167** | | P value |
| --- | --- | --- | --- | --- | --- | --- |
| Maternal age | | 32.00 (28.00–35.00) | | 33.00 (29.00–37.00) | | **0.005** |
| Weight | | 65.05 (58.35–76.10) | | 71.00 (61.00–84.00) | | **0.001** |
| BMI | | 24.96 (21.42–28.76) | | 27.02 (23.34–30.71) | | **<0.001** |
| Ethnicity | White | 175 | 56.8% | 74 | 44.3% | **0.002** |
|  | Black | 44 | 14.3% | 47 | 28.1% |  |
|  | Asian | 76 | 24.7% | 36 | 21.6% |  |
|  | Mixed, other | 13 | 4.2% | 10 | 6.0% |  |
| Nulliparity | | 171 | 55.5% | 89 | 53.3% | 0.642 |
| History of PTB | | 51 | 16.6% | 30 | 18.0% | 0.697 |
| Smoking | | 16 | 5.2% | 16 | 9.6% | 0.069 |
| ART Conception | | 16 | 5.2% | 8 | 4.8% | 0.848 |
| Chronic hypertension | | 4 | 1.3% | 8 | 4.8% | **0.030** |
| Diabetes mellitus | | 6 | 2.0% | 10 | 6.0% | **0.031** |
| PTB <34 | | 96 | 31.2% | 51 | 30.5% | 0.887 |
| MAP | | 86.30 (81.50–90.65) | | 89.50 (84.00–95.30) | | **<0.001** |
| UtA-PI | | 1.61 (1.25–1.97) | | 1.67 (1.26–2.07) | | **<0.001** |
| PAPPA_MoM | | 0.90 (0.64–  1.34) | | 0.89 (0.51–1.28) | | 0.270 |

Data are expressed as n (%) and median (IQR). BMI body mass index, PTB preterm birth, ART assisted reproductive technology, PE pre-eclampsia, MAP mean arterial pressure, UtA-PI uterine artery pulsatility index, PAPP-A pregnancy-associated plasma protein A, MoM multiple of median.

**Table S4.** Indications for iatrogenic preterm birth (PTB)

| **Indication for iatrogenic PTB** | **Number** | **%** |
| --- | --- | --- |
| Fetal growth restriction | 49 | 29.3 |
| Pre-eclampsia | 36 | 21.5 |
| Abnormal computerised cardiotocography | 23 | 13.8 |
| Maternal conditions | 23 | 13.8 |
| Stillbirth | 20 | 12.0 |
| Antepartum haemorrhage | 9 | 5.4 |
| Placenta previa/Placenta accreta spectrum disorders | 7 | 4.2 |
| **Total** | 167 | 100 |
